# Evaluation of the spraying of entomopathogenic nematode diluted in vinasse on *Stomoxys calcitrans* larvae (Diptera: Muscidae) under environmental conditions

**DOI:** 10.29374/2527-2179.bjvm001826

**Published:** 2026-04-13

**Authors:** Américo de Castro Monteiro, Ana Caroline Ferreira de Souza, Joana da Rocha Matos, Danielle Pereira da Silva, Gabriela Pereira Salça de Almeida, Vinícius Teixeira de Souza, Melissa Carvalho Machado do Couto Chambarelli, João Luiz Lopes Monteiro, Avelino José Bittencourt

**Affiliations:** 1 Programa de Pós-Graduação em Ciências Veterinárias, Departamento de Parasitologia Animal. Instituto de Veterinária, Universidade Federal Rural do Rio de Janeiro. Seropédica, RJ. Brazil.; 2 Departamento de Fitotecnia. Centro de Ciências Agrárias, Universidade Federal de Roraima. Cauamé, RR. Brazil.; 3 Departamento de Medicina e Cirurgia Veterinária, Instituto de Veterinária, Universidade Federal Rural do Rio de Janeiro. Seropédica, RJ, Brazil.

**Keywords:** sugarcane mill, biological control, fertigation, vinasse, flies, usina, controle biológico, fertirrigação, vinhoto, moscas

## Abstract

*Stomoxys calcitrans* is a hematophagous dipteran known as a "stable fly", capable of parasitizing several animal species. The use of biological agents is an alternative for controlling *S. calcitrans*, with entomopathogenic nematodes (EPNs) being both resistant and virulent. The present study aimed to evaluate the effect of infection by EPNs subjected to spraying pressure on *S. calcitrans* larvae in sugarcane straw and vinasse under environmental conditions. Groups of 20 larvae of the stable fly were placed in plastic containers containing one kilogram of autoclaved soil and 200 g of sugarcane straw each. Subsequently, 200 JIs/larvae were sprayed onto the trays with larvae, and emergence traps were placed on the containers. In the control group, there were no EPNs; only vinasse was used. The experiment had three replicates and was monitored daily for 15 days. It was observed that the EPN *Heterorhabditis bacteriophora* HP88 showed a mortality rate of 33.3%, which was not statistically different from that of the control group (38.3%). The EPNs *H. indica LPP30* and *H. baujardi LPP7* caused mortality rates of 81.7% and 73.3%, respectively; both being higher than *H. bacteriophora HP88* and the control group, but equal to each other under these conditions. It is concluded that *H. indica* LPP30 and *H. baujardi* LPP7 were effective against stable fly larvae under environmental conditions, whereas *H. bacteriophora* HP88 does not demonstrate similar efficacy.

## Resumo

*Stomoxys calcitrans* é um díptero hematófago conhecido como “mosca-dos-estábulos”, capaz de parasitar diversas espécies animais. O uso de agentes biológicos é uma alternativa no controle de *S. calcitrans*, com os nematoides entomopatogênicos (NEPs) sendo seres resistentes e virulentos. O presente estudo objetivou avaliar o efeito da infecção de NEPs submetidos à pressão de pulverização sobre larvas de *S. calcitrans* em palha de cana-de-açúcar e vinhaça no ambiente. Grupos de 20 larvas da mosca-dos-estábulos foram depositados em recipientes plásticos contendo um quilo de solo autoclavado e 200 g de palha de cana, cada. Em seguida pulverizou-se 200 JIs/larva sobre bandejas com larvas, então foram colocadas armadilhas de emergência sobre os recipientes. No grupo controle não havia NEPs, apenas vinhaça. O experimento teve três repetições e foi observado diariamente durante 15 dias. Observou-se que o NEP *Heterorhabditis bacteriophora* HP88 apresentou taxa de mortalidade de 33,3%, não havendo diferença estatística para grupo controle (38,3%). Já os NEPs *H. indica* LPP30 e *H. baujardi* LPP7 causaram taxas de mortalidade de 81,7% e 73,3%, respectivamente; sendo ambos superiores a *H. bacteriophora* HP88 e ao grupo controle, porém iguais entre si nessas condições. Conclui-se que em condições ambientais, *H. indica* LPP30 e *H. baujardi* LPP7 foram eficazes contra larvas da mosca-dos-estábulos, enquanto *H. bacteriophora* HP88 não apresentou semelhante eficácia.

## Introduction

*Stomoxys calcitrans* (Linnaeus, 1758) is a hematophagous dipteran known as "stable fly", capable of parasitizing several animal species, such as cattle, horses, sheep, goats, pigs, dogs, cats, and can also feed on wild animals, birds, and even humans (Bittencourt, 2012).

The stable fly has a cosmopolitan distribution, with population increases during the hottest periods of the year (Bittencourt, 2012). The seasonality of *S. calcitrans* shows two annual population peaks in tropical and subtropical countries, associated with warmer and wetter periods, although the fly can persist year-round (Mullens & Meyer, 1987). Dominghetti et al. (2015), in the Brazilian Central Plateau, reported a greater abundance of the insect between April/May and December, when temperatures ranged from 18.6 to 26.8ºC, a period when sugarcane harvest occurs.

Both males and females are hematophagous, causing severe blood spoliation and serving as vectors for several pathogens (Castro et al., 2008). All these factors lead to considerable economic losses, with estimates of US $2.221 billion in the United States (Taylor et al., 2012) and US $335.5 million per year in Brazil (Grisi et al., 2014). It should be noted that these values are underestimated, as they do not account for recent outbreaks of this insect, especially in the Midwest and Southeast regions of Brazil (Souza et al., 2021).

The fly’s behavior during outbreaks has forced cattle owners to make changes in cattle management and transport the animals to areas free of this insect. These factors have led many people to abandon the livestock activity, and have also fostered an intense and warm discussion between livestock farmers and the sugar and alcohol industry (Bittencourt, 2012).

According to Companhia Nacional de Abastecimento (2025), the 2025/26 sugarcane harvest produced an estimated 666.4 million tons across more than 8.9 million cultivated hectares, yielding 45 million tons of sugar and 27.37 billion liters of ethanol. This amount resulted in approximately 480 billion liters of vinasse, 26 million tons of filter cake, and 1.2 million tons of ash, while the amount of sugarcane straw deposited on the soil varies between 8 and 20 tons per hectare (Menandro et al., 2017).

Among the sugarcane by-products, straw with vinasse and filter cake are the ones with the greatest potential to cause population explosions of the stable fly, with the filter cake being the substrate with the highest production of flies per square meter (55.8 flies/m^2^). Fortunately, the filter cake is produced in smaller amounts compared to straw with vinasse, which produces 24.2 flies/m^2^, making the latter two the most important in the outbreak dynamics due to the large extent of fertigated areas (Corrêa et al., 2013).

The use of biological agents is an alternative for controlling *S. calcitrans*, with several studies conducted under controlled laboratory conditions. Ratcliffe et al. (2002) observed promising results using parasitoid Hymenoptera in the control of immature stages of *S. calcitrans*. Conversely, Moraes et al. (2015) reported that the entomopathogenic fungus *Beauveria bassiana* was not effective in controlling immature stages of the stable fly. Monteiro-Sobrinho et al. (2025) and Leal et al. (2017) used entomopathogenic nematodes of the genus *Heterorhabditis* to control *S. calcitrans*, in which these organisms were able to cause high larval mortality rates.

In Brazil, the use of EPNs has been studied mainly for the control of arthropod pests in agriculture, with high mortality rates (Leite et al., 2003). In the past decade, Monteiro et al. (2014) took an important step towards controlling pests that affect domestic animals by employing EPNs to target the tick *Rhipicephalus* (Boophilus) *microplus* using an insect-cadaver formulation (*Galleria mellonella*). Subsequently, Monteiro-Sobrinho et al. (2016) and Leal et al. (2017) initiated studies in Brazil on the control of *S. calcitrans* with entomopathogenic nematodes.

The present study aimed to evaluate the effect of infection by the EPNs *Heterorhabditis bacteriophora* HP88, *H. baujardi* LPP7, and *H. indica* LPP30 subjected to spraying pressure on *S. calcitrans* larvae in sugarcane straw and vinasse under environmental conditions.

## Materials and methods

### Maintenance of the *Stomoxys calcitrans* colony to obtain larvae

The adult dipterans were captured on the UFRRJ campus with an entomological net, stored in plastic cages (15x15x20cm) for transport, transported to the laboratory, and identified according to Furman and Catts (1982). The flies were kept in plastic cages (covered with Nylon® mesh) of larger dimensions (60x40x50cm) and fed with bovine blood. The blood used in the fly diet was obtained from cattle slaughtered at a slaughterhouse in the municipality of Barra Mansa - RJ, to which 0.38% sodium citrate was added (Watson et al., 1995).

Eggs were collected directly from the pads, where blood was deposited to feed the flies, and a black fabric extended over the cages, washed in running water, and collected in a 500-mesh particle-size sieve. The medium developed by Christmas (1970) and adapted by Barros et al. (2014) was used to obtain larvae, consisting of sugarcane (330g), wheat bran (125g), meat meal (40g), sodium bicarbonate (5g), and distilled water (125mL). The colony of flies was maintained at 27±1 °C and 70% - 80% relative humidity.

### Maintenance of the entomopathogenic nematode colony

The nematodes come from the nematode collection at the Universidade do Norte Fluminense (UENF).

The nematodes used were maintained and multiplied through *in vivo* multiplication in *Galleria mellonella* and *Tenebrio molitor* (Lindegren et al., 1993; Kaya & Stock, 1997). Infective juveniles (IJs) were stored in a B.O.D. incubator chamber (Eletrolab®, EL 212 model) at 16 ± 1 °C and 70% - 80% RH in 40mL cell culture flasks. The nematodes used in the experiments described below were not stored in a climate chamber; they were captured directly from White's traps (White, 1927) and used immediately after being collected.

### Quantification of entomopathogenic nematodes suspensions

IJs were quantified by counting 12 aliquots of ten μL obtained from an aqueous suspension of EPNs. Then they were removed from the traps and placed in a 40 mL cell culture bottle. After counting the IJs in the 12 aliquots, the highest and lowest amount of EPNs/aliquot were discarded, and the average number of IJs was calculated from the remaining ten aliquots. From this calculation, the concentration of the suspensions was adjusted to IJs/mL (Taylor et al., 1998). The dosage of nematodes and pressure was chosen according to the findings of Monteiro-Sobrinho et al. (2025).

### Pressurization system

**Figure 1:**
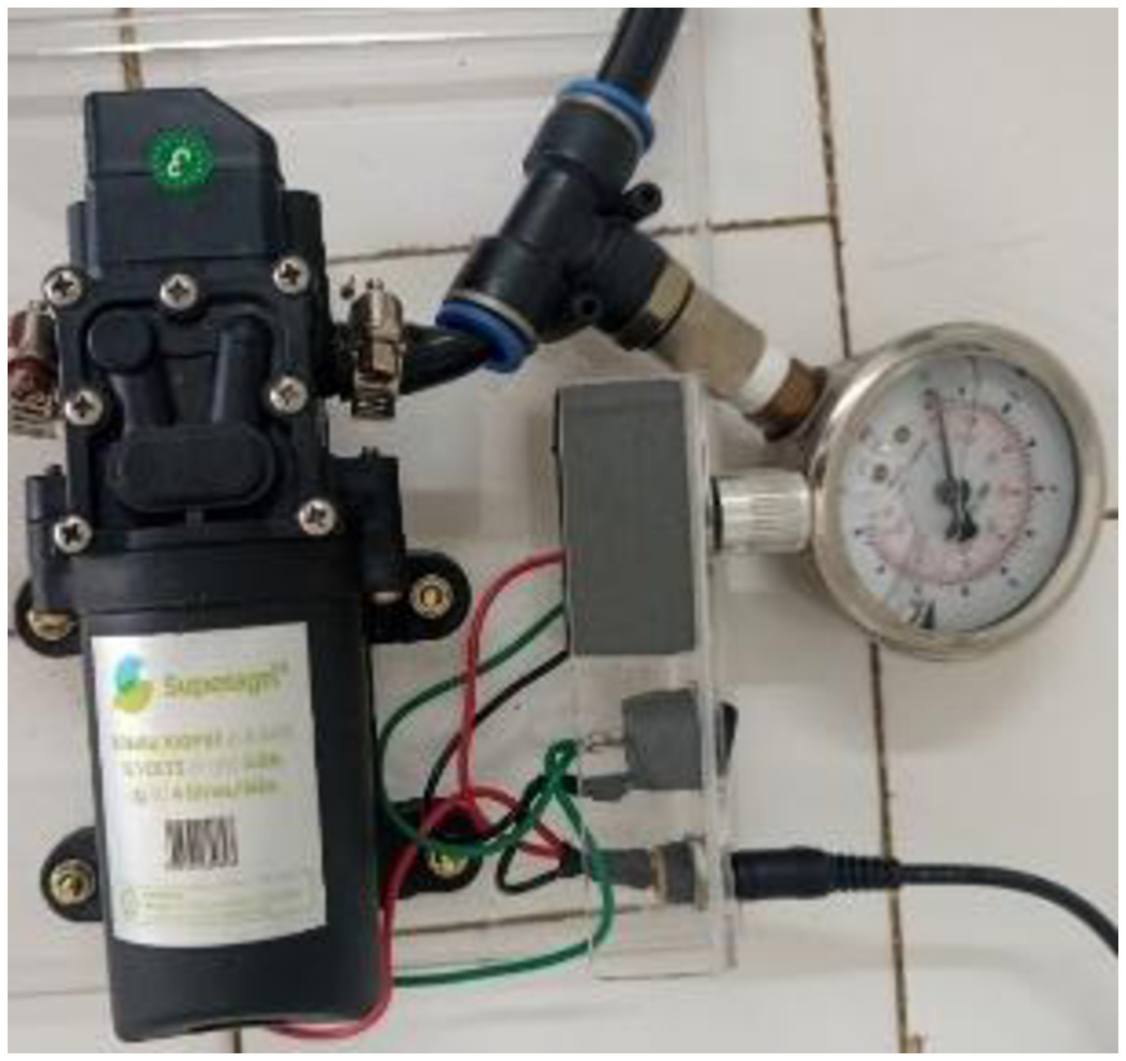
Hydraulic pump with manometer and pressure controller.

The pressure used in this study was similar to that generated by spray and aspersion systems used for fertigation in sugarcane fields in Brazil (ranging between 58 and 71 psi) (Testezlaf, 2017). To obtain the desired pressure (60 psi), a hydraulic pump (Superagri ®, 100 psi, 12 volts, 3.0 amps, 4 L/min flow) with a pressure controller and a spray nozzle was used. A manometer was attached to this pump for pressure measurement (Figure 1).

### Spraying of entomopathogenic nematodes onto *Stomoxys calcitrans* larvae in plastic trays maintained under environmental conditions

**Figure 2:**
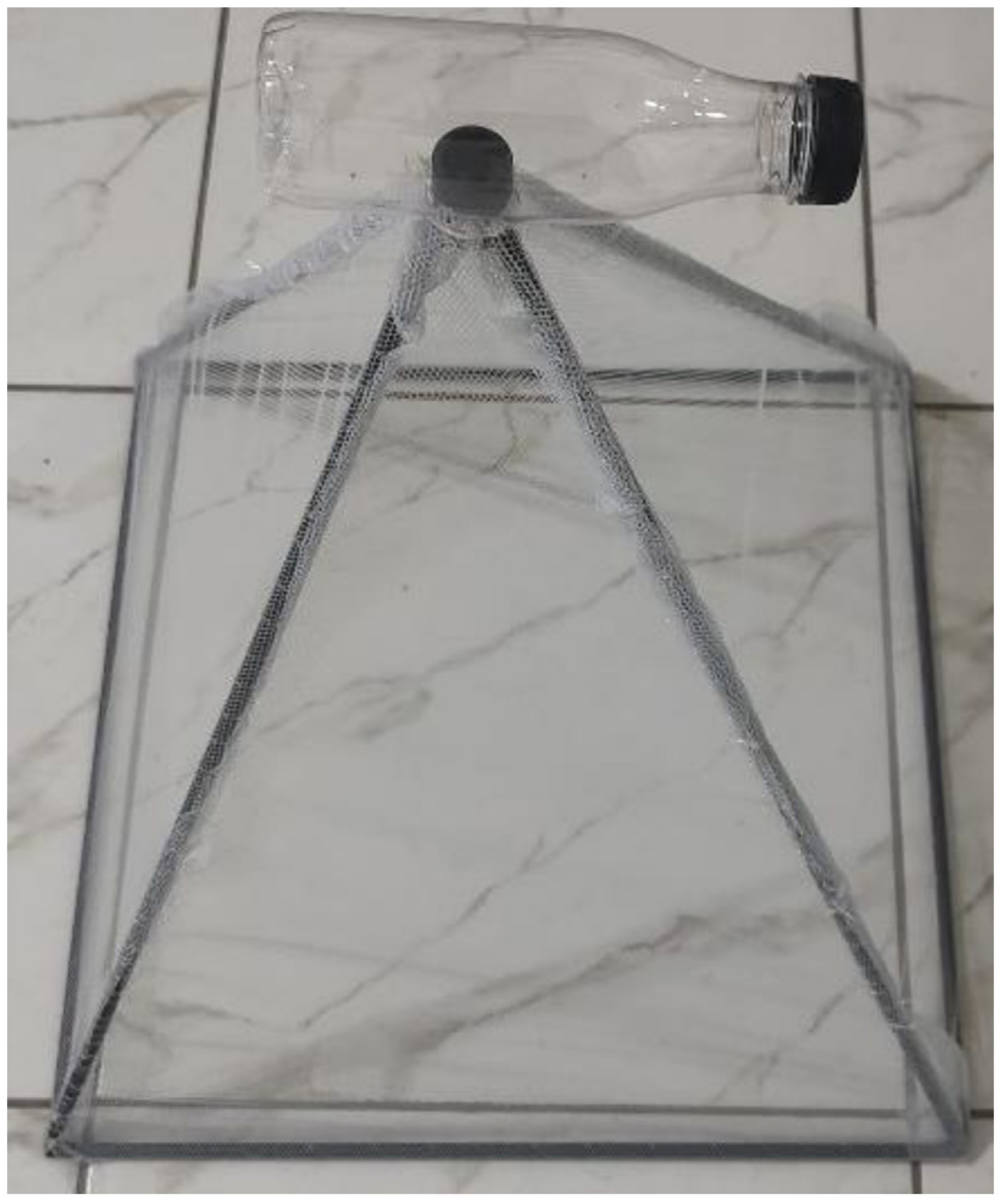
Emergence trap with plastic collector.

The emergence trap model used in the present study was provided by researchers from Embrapa gado de corte, Dr. Antonio Thadeu Medeiros de Barros and Dr. Paulo Henrique Duarte Cançado (communication via the internet); it was based on that of Corrêa et al. (2013). The traps consisted of a pyramidal metal frame (50 x 50 x 50 cm) and were covered with translucent fabric (tulle). A metal ring was welded at the top of the pyramidal frame to hold a 400 mL plastic bottle (collector) for capturing newly emerged flies (Figure 2).

**Figure 3:**
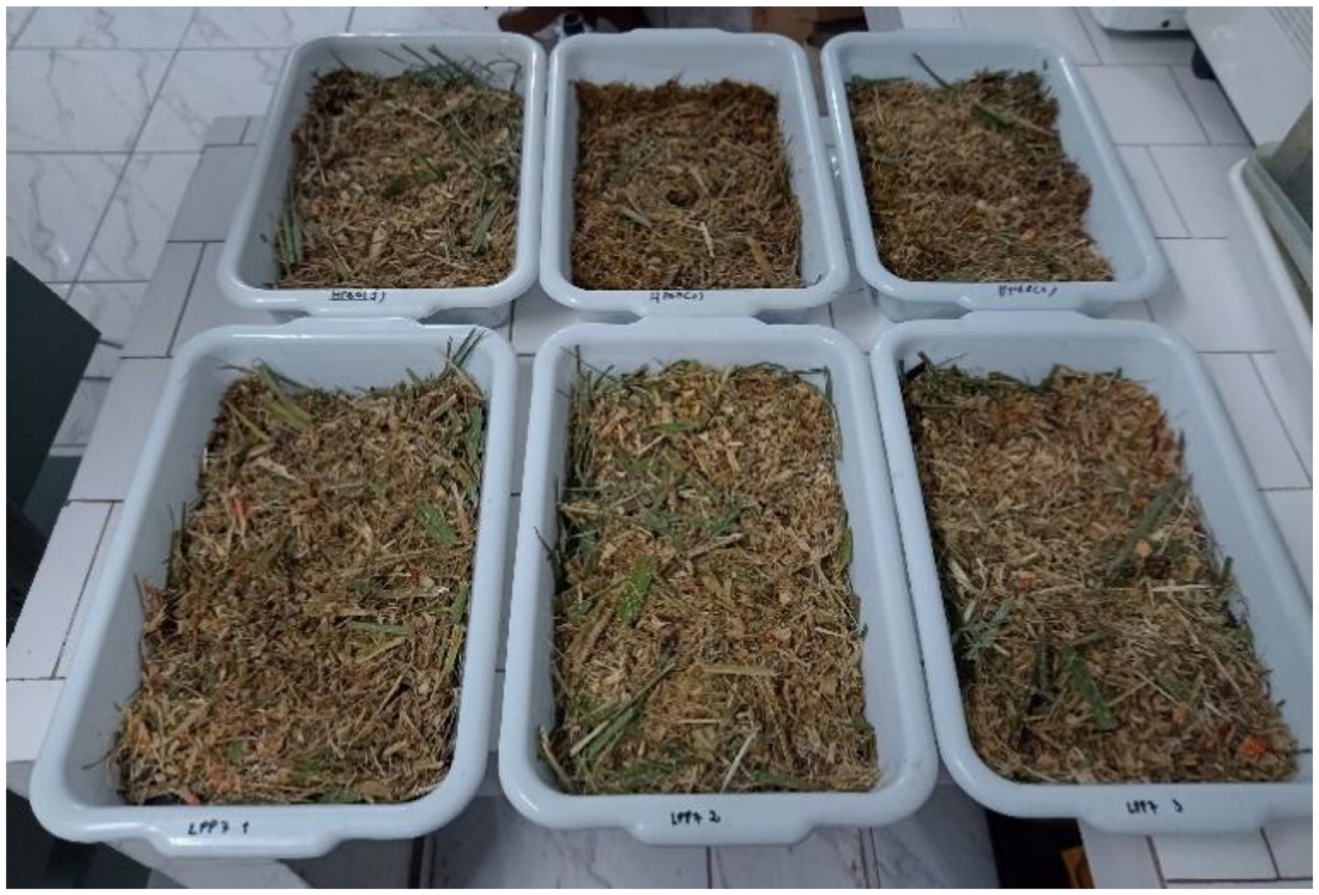
Plastic containers containing soil and sugarcane straw.

**Figure 4:**
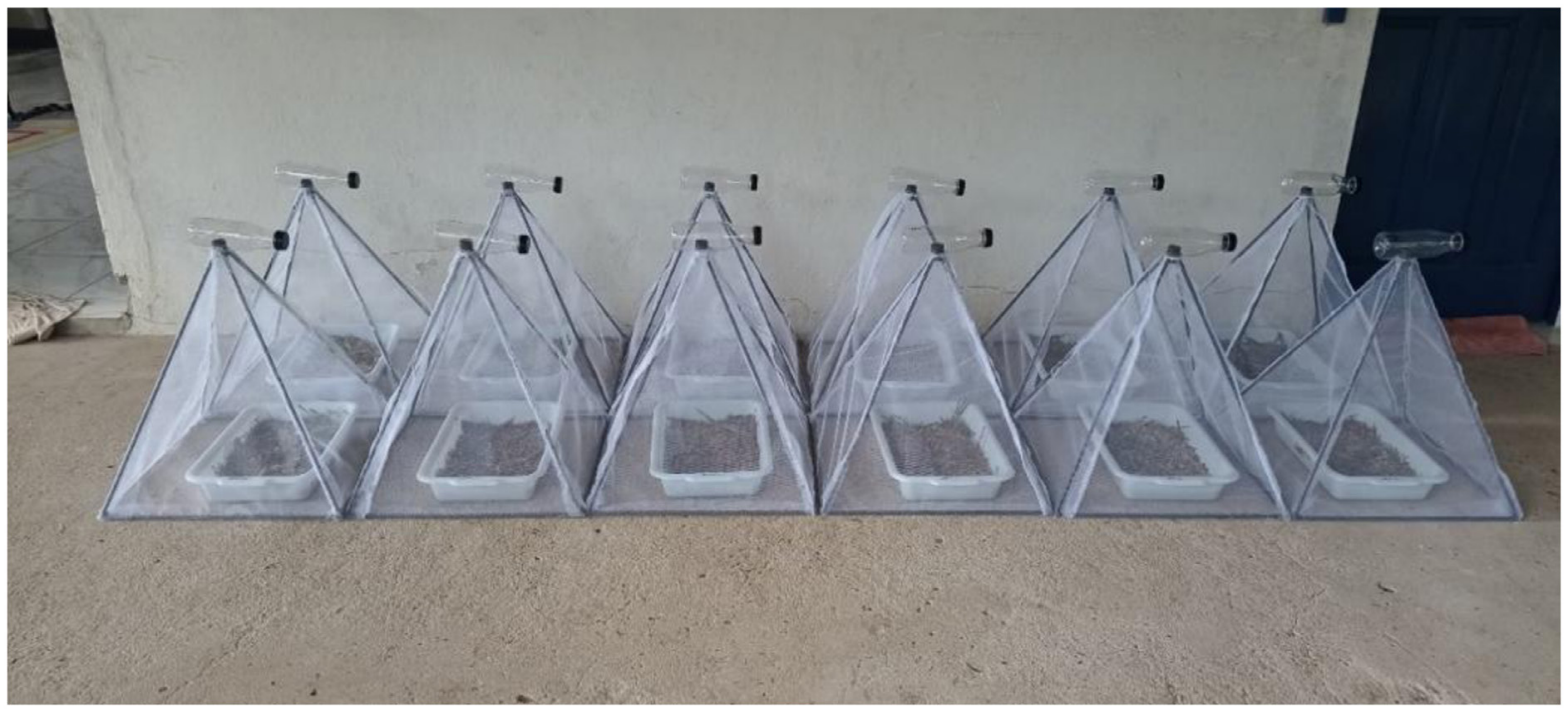
Emergence traps on plastic trays containing soil, sugarcane straw, and *Stomoxys calcitrans* larvae exposed to entomopathogenic nematodes sprayed in vinasse.

Groups of 20 third-instar stable fly larvae (eight to ten days old) were placed with entomological forceps into plastic containers (35 x 20 x 10 cm), each containing one kilogram of autoclaved soil and 200 g of sugarcane straw (Figure 3). Next, 200 IJs/larvae (diluted in 450 mL of 50% vinasse) were sprayed onto the trays with larvae. Emergence traps were then placed on the containers to observe possible adult fly emergence (Figure 4). In the control group, there were no EPNs; only vinasse was used. IJs of *H. bacteriophora HP88*, *H. indica LPP30,* and *H. baujardi LPP7* in vinasse solution (heated to 35 ºC) were applied under pressure of 60 psi. The experiment was conducted outdoors, under environmental conditions, but in a shady location; it had three replicates and was observed daily for 15 days.

### Experimental design and statistical analysis

The data were analyzed using the Shapiro-Wilk test for normality and Bartlett’s test for homoscedasticity. After the assumptions were met, an analysis of variance (ANOVA) was performed, followed by Tukey test (*p* <0.05) to compare the means among groups. Statistical analyses and graphs were performed using GraphPad Prism 9.5.1.

## Results

**Figure 5:**
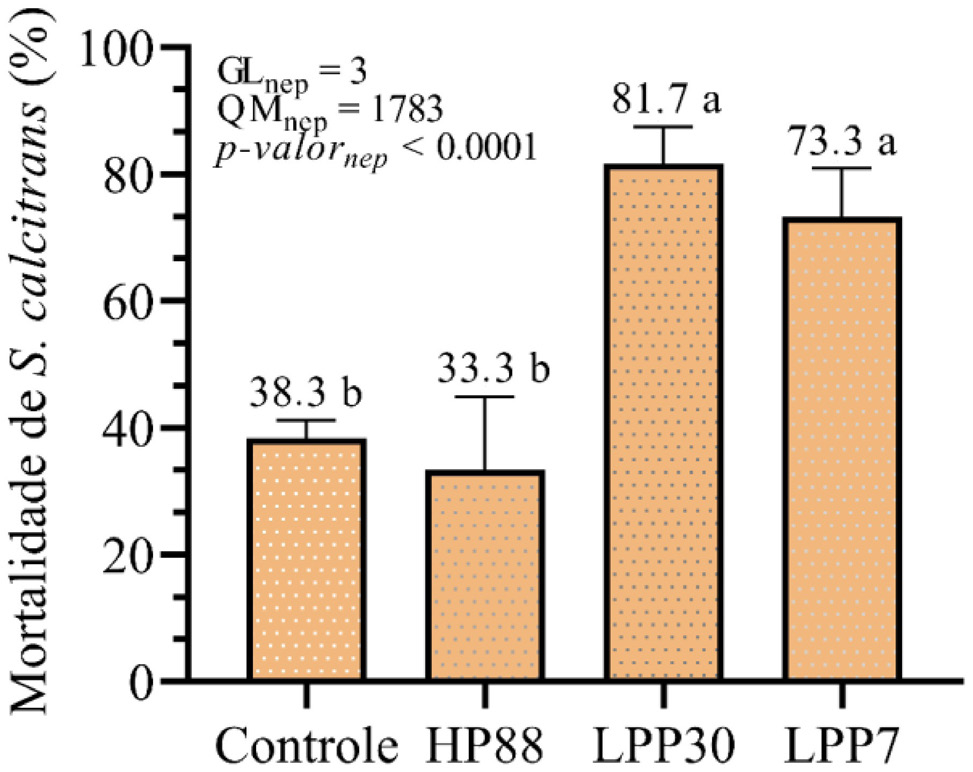
Larval mortality of *Stomoxys calcitrans* in sugarcane straw caused by *Heterorhabditis bacteriophora* HP88, *H. indica* LPP30, and *H. baujardi* LPP7. Means followed by the same letter do not differ from each other according to Tukey test (P <0.05).

The EPN *H. bacteriophora* HP88 showed a mortality rate of 33.3% when sprayed with vinasse at 60 psi onto trays containing stable fly larvae in sugarcane straw under environmental conditions, which was not statistically different from the control group (38.3%). The EPNs *H. indica LPP30* and *H. baujardi LPP7* caused mortality rates of 81.7% and 73.3%, respectively; both higher than those observed for *H. bacteriophora HP88* and the control group, but equal to each other under these conditions (Figure 5).

### Discussion

EPNs are highly resistant and virulent; even after exposure to temperatures exceeding 30 ºC and when applied in vinasse, they caused larval mortality of *S. calcitrans* above 90%. In addition, they were able to search for and kill fly larvae in various substrates associated with sugarcane activity (Monteiro-Sobrinho et al., 2023).

In Brazil, three EPN-based products are already registered. Recently, the company Koopert do Brasil Holding S.A. began marketing a product containing EPNs of the species *Steinernema carpocapsae*, recommended for the control of agricultural pests. The sugarcane weevil, *Sphenophorus levis* (Coleoptera: Curculionidae), is among the pests. This opens a great possibility to employ EPNs in the sugarcane field to control the stable fly, as the use of these organisms in sugarcane crops is not new.

The frequent, improper and indiscriminate use of chemical insecticides for parasite control has reduced the efficacy of these substances, particularly those of the pyrethroid class. This reality has been evidenced by the emergence of resistant individuals in different fly populations, especially in houseflies (*Musca domestica*), horn flies (*Haematobia irritans*), and stable flies (Barros et al., 2019). Consequently, it demands new methods to control these pests (Davidson & Sweeney, 1983), as observed in the present study.

**Figure 6:**
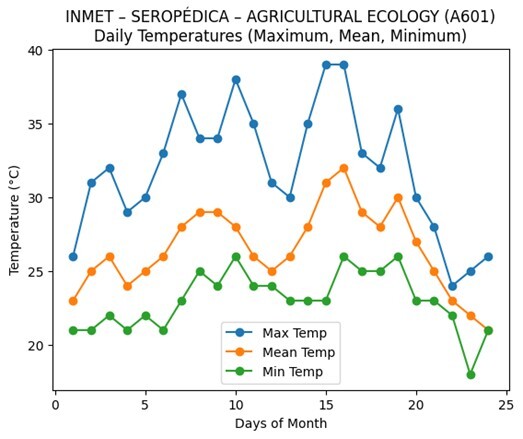
Temperature graph in the city of Seropédica during January 2024. Source: National Institute of Meteorology (Instituto Nacional de Meteorologia, 2024).

**Figure 7:**
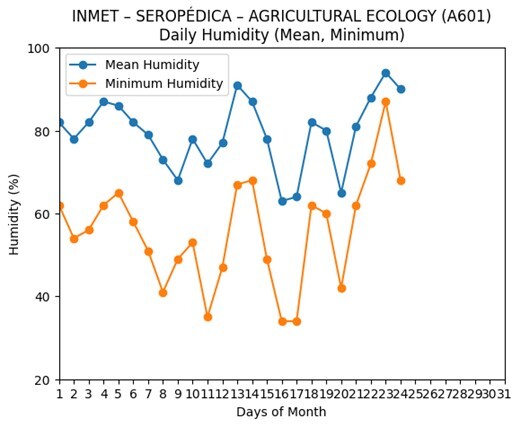
Humidity graph in the city of Seropédica during January 2024. Source: National Institute of Meteorology (Instituto Nacional de Meteorologia, 2024).

A spraying pressure of 60 psi was chosen for this study because, under laboratory conditions, EPNs subjected to this pressure did not show a drop in their effectiveness (Monteiro-Sobrinho et al., 2025). When EPNs were evaluated under environmental conditions, it was observed that the virulence of *H. bacteriophora* HP88 decreased considerably compared to the findings of Monteiro-Sobrinho et al. (2025), in which larval mortality reached 90%. In contrast, the present study found that mortality of *S. calcitrans* larvae caused by the same EPN species was 33.3% (Figure 5). This result is considerably lower than those observed under laboratory conditions by Monteiro-Sobrinho et al. (2025), which may have occurred due to the large variation in temperature and humidity during the 15-day experimental period (January 11 to 26, 2024), when temperatures in the city of Seropédica-RJ reached peaks of approximately 40 ºC (Instituto Nacional de Meteorologia, 2024) (Figure 6), and relative humidity ranged from around 60 to 95%, with minimum values of approximately 35% (Instituto Nacional de Meteorologia, 2024) (Figure 7).

The factors described above may have negatively affected the action of *H. bacteriophora* HP88. This EPN apparently did not withstand the high temperatures and humidity variations typical of the summer in the Baixada Fluminense region in Rio de Janeiro. The temperature was probably the most important and impactful factor, as the experiment contained vinasse, thereby maintaining the necessary humidity for the nematodes. Monteiro-Sobrinho et al. (2023) reported that *H. bacteriophora* HP88 was able to cause an average mortality rate of 97.8% in stable fly larvae under laboratory conditions with temperatures ranging from 16 to 35 ºC and 50% vinasse. This may be explained by the origin of this nematode in the State of New Jersey, USA (Alves et al., 2009), a temperate climate region, indicating that this agent is more effective at temperatures equal to or below 35 ºC.

In fact, the main limitation to implementing control programs using EPNs in tropical regions is their sensitivity to high temperatures, which alter mobility, survival, development, reproduction, and infection capacity of these organisms (Dunphy & Webster, 1986). According to Mukuka et al. (2009) and Ulu and Susurluk (2015), *H. bacteriophora* can be severely damaged at temperatures close to or above 40 ºC. Therefore, it is important to research and use native EPN species, as they are adapted to the local climatic conditions (Dolinski & Moino Junior, 2006).

Unlike *H. bacteriophora HP88*, the EPNs *H. indica* LPP30 and *H. baujardi* LPP7 are native to the Brazilian territory, with *H. indica* LPP30 coming from Campos dos Goytacazes, Rio de Janeiro; whereas *H. baujardi* LPP7 is native to the Amazon forest, having been isolated in Monte Negro, Rondonia (Dolinski et al., 2008).

*H. indica* LPP30 and *H. baujardi* LPP7 caused high mortality rates in *S. calcitrans* larvae under environmental conditions (Figure 5). These nematodes presented mortality rates close to those found in the bioassays by Monteiro-Sobrinho et al. (2025), indicating that these species apparently did not suffer from the high temperatures and humidity variations that affected the State of Rio de Janeiro in January 2024. These temperatures are very similar to those observed during the summer in the Central-West region of Brazil (Marcuzzo et al., 2012), where stable fly outbreaks have been frequent in recent decades (Barros et al., 2023). This demonstrates that, in the future, *H. indica* LPP30 and *H. baujardi* LPP7 may be used in *S. calcitrans* control programs, even during the hottest periods of the year.

## Conclusions

It is concluded that *H. indica* LPP30 and *H. baujardi* LPP7 are effective against stable fly larvae under environmental conditions, whereas *H. bacteriophora* HP88 does not exhibit similar efficacy.
